# Aged blood factors decrease cellular responses associated with delayed gingival wound repair

**DOI:** 10.1371/journal.pone.0184189

**Published:** 2017-09-12

**Authors:** María Paz Saldías, Christian Fernández, Alejandra Morgan, Catalina Díaz, Diego Morales, Fabián Jaña, Alvaro Gómez, Alonso Silva, Fernanda Briceño, Alejandro Oyarzún, Felipe Maldonado, Oscar Cerda, Patricio C. Smith, Mónica Cáceres

**Affiliations:** 1 Program of Molecular and Cell Biology, Institute of Biomedical Sciences, Faculty of Medicine, Universidad de Chile, Santiago, Chile; 2 Universidad de Aysén, Coyhaique, Chile; 3 Faculty of Dentistry, Universidad Finis Terrae, Santiago, Chile; 4 Department of Anesthesia, Faculty of Medicine, Hospital Clínico de la Universidad de Chile, Santiago, Chile; 5 Millennium Nucleus of Ion Channels-Associated Diseases (MiNICAD), Santiago, Chile; 6 School of Dentistry, Faculty of Medicine, Pontificia Universidad Católica de Chile, Santiago, Chile; Boston University Henry M Goldman School of Dental Medicine, UNITED STATES

## Abstract

Aging is a gradual biological process characterized by a decrease in cell and organism functions. Gingival wound healing is one of the impaired processes found in old rats. Here, we studied the *in vivo* wound healing process using a gingival repair rat model and an *in vitro* model using human gingival fibroblast for cellular responses associated to wound healing. To do that, we evaluated cell proliferation of both epithelial and connective tissue cells in gingival wounds and found decreased of Ki67 nuclear staining in old rats when compared to their young counterparts. We next evaluated cellular responses of primary gingival fibroblast obtained from young subjects in the presence human blood serum of individuals of different ages. Eighteen to sixty five years old masculine donors were classified into 3 groups: “young” from 18 to 22 years old, “middle-aged” from 30 to 48 years old and “aged” over 50 years old. Cell proliferation, measured through immunofluorescence for Ki67 and flow cytometry for DNA content, was decreased when middle-aged and aged serum was added to gingival fibroblast compared to young serum. Myofibroblastic differentiation, measured through alpha-smooth muscle actin (α-SMA), was stimulated with young but not middle-aged or aged serum both the protein levels and incorporation of α-SMA into actin stress fibers. High levels of PDGF, VEGF, IL-6R were detected in blood serum from young subjects when compared to middle-aged and aged donors. In addition, the pro-inflammatory cytokines MCP-1 and TNF were increased in the serum of aged donors. In old rat wound there is an increased of staining for TNF compared to young wound. Moreover, healthy gingiva (non injury) shows less staining compared to a wound site, suggesting a role in wound healing. Moreover, serum from middle-aged and aged donors was able to stimulate cellular senescence in young cells as determined by the expression of senescence associated beta-galactosidase and histone H2A.X phosphorylated at Ser139. Moreover, we detected an increased frequency of γ-H2A.X-positive cells in aged rat gingival tissues. The present study suggests that serum factors present in middle-aged and aged individuals may be responsible, at least in part, for the altered responses observed during wound healing in aging.

## Introduction

The proportion of people over 60 years old is growing faster than any other age group. By 2050, the number of people over 60 years is projected to be five times than in 1950 [1]. Aging is associated with a decline in health, and altered gingival wound healing is one of the impaired processes found during aging [2].

During wound healing, coagulation is activated through the formation of a fibrin clot that serves as a temporary matrix, providing a mesh of fibrin and a reservoir of cytokines and growth factors like PDGF, TGFβ1 and 2, EGF, VEGF, HGF, FGF-2, RANTES, MIP-1α and IL-8 [3, 4]. This group of early growth factors provides chemotactic cues to recruit inflammatory cells into the wound site, initiates reepithelization and the migration and proliferation/activation of fibroblasts [3]. Three to five days after injury, the formation of the granulation tissue begins, with the migration and activation of fibroblasts, endothelial cells and macrophages into the blood clot [5]. About 7 to 10 days after wounding, the granulation tissue becomes hypercellular containing abundant fibroblasts and endothelial cells. During this process fibroblasts are differentiated into active myofibroblasts, a transient cell population involved in the synthesis and organization of the newly formed extracellular matrix and wound contraction [5]. Importantly, myofibroblast differentiation is important for matrix deposition and organization during wound healing. To do that, myofibroblast express a new actin isoform known as α smooth muscle actin that reinforces the wound remodeling capacity of these cells [6]. Using the parabiosis animal model, that connects the circulatory system of young and old animals, previous studies have described an improvement in the repair of aged bone fracture [7] and muscle lesions in aged conditions [8] and reversion of age related impairments in cognitive functions and synaptic plasticity in mice [9]. These studies have suggested that growth factors present in the blood of aged subjects may play a detrimental role in wound healing. Interestingly, the role of blood-derived factors in oral wound healing remains unknown.

The present study was designed to evaluate whether factors present in the serum of aged and middle-aged subjects may partially reproduce delayed wound-healing responses associated with aging.

## Material and methods

### Cell culture

Primary cultures of human gingival fibroblasts (HGF) were obtained from healthy gingival tissue surrounding bicuspids obtained from non-smoking, systemically healthy individuals. Periodontal examination demonstrated sites with probing depth <4 mm, no loss of attachment and no bleeding on probing. Primary cultures of Human Gingival Fibroblast (GF) were established by the explant method and were cultured in DMEM (Gibco BRL, Grand Island, NY) containing 10% fetal bovine serum (FBS) (Hyclone laboratories Inc, Logan, Utah) and antibiotics as previously described [10]. Tissue explants were obtained from clinically healthy gingiva of 5 individuals (15–25 years old) after the extraction of third molar. Tissue samples were harvested with the informed consent of the patients. The protocol for tissue obtainment was approved by the Ethical Committee, Faculty of Medicine, Universidad de Chile (095–2014). All experiments were performed using cells expanded between passages three and nine.

Serum collection was acquired by coagulating 10 mL of blood at 37°C for 1 hour and then centrifuged at 2500 rpm during 10 min. The supernatant corresponds to serum. We used serum from 45 non-smoker healthy male volunteers from different ages (18 to 70 years old), with no acute or chronic infectious diseases, or any clinical disorders or chronic drug intake. All donors signed an informed consent in agreement to the Ethical Committee, Faculty of Medicine, Universidad de Chile. All these procedures were performed in accordance to the protocol 095–2014. None of the donors were from a vulnerable population and all donors provided written informed consent that was freely given. We defined three groups of study: “young” (between 18 and 22 years old) (n = 21), “middle-aged” 30–48 years old (yo) (n = 13) and “aged” or “elderly” (50 or more years old) (n = 11). The average and standard deviation of age of these 3 group was 20.16 ±1,51 young, 34.83 ± 5.98 middle-aged and 57.578 ± 5.25 aged years old respectively.

#### Wound healing studies

Five young (2 months old) and five old (18 months old) male Sprague-Dawley rats were used for wound healing studies, Full-thickness excisional wounds were made in the palatal gingiva close to the first and second upper molars (1.0 mm x 3 mm) with the aid of a periodontal probe (Hu-Friedy) as previously described [2]. Animal procedures were conducted in accordance to the Administrative Panel on Laboratory Animal Care at Universidad de Chile (CBA 0706) and following the ARRIVE (Animal Research Reporting of *in vivo* Experiments) guidelines for animal experimentation [11]. During aging of the rats and the wound healing assay we maintained 2 rats for cage with light/dark cycle and food at libitum. After 7 days of wound repair, rats were anesthetized with 2% isofluorane administered using a nasal mask and then animals were sacrificed by an intracardiac euthanasia (T61) that contained (Embutramide 0.2 g, Mebezonium iodide 0.05 g y Tetracaine hydrochloride 0,005g for 1 mL)

Tissues were fixed in 10% buffered formalin, decalcified in 10% formic acid and were stained with Masson Trichrome and scanned using a Nanozoomer -XR digital slide scanner C12000 (Hamamatsu Photonics KK, Hammamatu city, Japan). For immunostaining, 5μm cross sections were treated with Tris-EDTA pH = 9 for 30 min at 80°C, blocked during 2 h with 10% calf serum and Triton-X100 0,3%, incubated with anti-Ki67 (D3B5,Cell Signaling technology, Danvers, MA, USA) and anti-vimentin (V6389,Sigma, St. Louis, MO, USA) antibodies. Primary antibodies were detected with anti rabbit IgG(H+L) and anti mouse-IgG1 conjugated with alexa fluor 488 and alexa fluor 555 secondary antibodies, respectively (Invitrogen, Carlsbad, CA, USA). Images were acquired with a spinning disk Olympus IX81 microscope. No primary antibody was used as a negative control. Nuclei were stained with Hoechst.

For TNF staining and phospho Histone H2A.X (Ser139) we treated 5μm cross section with buffer citrate (10mM citric acid, 0,05% Tween-20, pH = 6), for 30 minutes at 90°C. Then, treated during 20 minutes with H_2_O_2_ at 3%v/v. Blocked with 10% calf serum and 0,3%Triton X-100 during 2 hours, incubated with anti TNF (ab6671, Abcam, Cambridge, MA,USA) or pSer139 H2A.X (clone JBW301,Merck) overnight at 4°C. In negative control sections, primary antibody was replaced by non immune calf serum. Primary antibody was detected using RTU biotinylated antibody universal and mouse/rabbit IgG during 30 minutes at room temperature (BP-1400, Vector Laboratories, Burlingame, CA, USA). Then, we used Vectastain Elite ABC kit system (PK-6100, Vector, Burlingame, CA, USA) during 30 minutes at room temperature. After 3 washed, immunoreactive signal was detected with DAB Peroxidase substrate (SK-4100, Vector Laboratories). After immunohystochemical procedures, Hematoxilyn Delafield was used for counterstaining.

To quantify wound closure tissue sections were stained with Masson trichrome and images were scanned using a Nanozoomer digital slide scanner and analyzed by the NDP.view software version 5.0.4.1382. This software allows us to quantify the area of the granulation tissue expressed as mm^2^. The amount of collagen (detected through the Masson Trichrome stain) was quantified for each specimen by measuring the area of collagen as μm^2^ in the granulation tissue (area of the cells were excluded) and expressed as percentage of the total area of granulation tissue. For TNF stained we selected at least 5 ROI of 55 μm^2^ in the wound area, in this selection area we obtained the minimal intensity and the mean intensity of the chromogenic stain.

1 mL of blood were coagulated at 37°C for 1 hour and then centrifuged at 2500 rpm during 10 min. The supernatant correspond to the blood serum, aliquots were stored at -80°C until use. Rat blood serum cytokines were evaluated using rat cytokine profile antibody array (ARY 008, R&D system Minneapolis MN, USA).

### Immunofluorescence

Cells were grown on coverslips, fixed with 4% (w/v) paraformaldehyde, blocked, and incubated with primary anti Ki67 antibody (D3B5,Cell Signaling technology, Danvers, MA, USA) used as a marker for proliferation and alpha-Smooth Muscle Actin alpha (α-SMA) (A2547,Sigma, St. Louis, MO, USA) as myofibroblasts differentiation marker. Primary antibodies were detected with Alexa-conjugated secondary antibodies (Invitrogen). F-actin was stained with Alexa-Fluor-555-phalloidin (Invitrogen Molecular Probes, Carlsbad, CA, USA) and DNA was stained with Hoechst (Sigma, St. Louis, MO, USA). Images were acquired with a spinning disk Olympus IX81 microscope.

### Cell cycle analysis

Fifty thousand cells were incubated in DMEM for 16h. Then, cells were treated with 10% (v/v) serum from volunteers of different ages for 24 h. Cells suspensions were fixed with methanol overnight. Samples were centrifuged and incubated with 40 μg/mL propidium iodide (Sigma, St. Louis, MO, USA) and RNAase A (100 μg/mL). Cells were then analyzed by flow cytometer (FACSCanto, Becton Dickinson)

#### Quantification of blood factors

To evaluate MCP-1, PDGF, HGF, TNF, VEGF and IL-6R, serum samples were diluted 5 times and quantified by ELISA multiplex array (Quansys, biosciences). TGFβ1 was diluted 5 times in PBS, treated with HCl 1M during 10 min, neutralized with NaOH 1N and analyzed by ELISA affymetrix (eBioscience, San Diego, CA, USA.). Cholesterol was measured with an enzymatic method Colestat liquid line (Wiener lab, Rosario, Argentina).

### Western blot

GF were incubated during 72 h with 10% (v/v) serum from volunteers of different ages. Cells were lysed with a buffer containing 50 mM Hepes pH 7.4, 150 mM NaCl, 2 mM MgCl_2_, 2 mM EGTA, 1% v/v Triton X-100, 10% v/v glycerol, 2 mM PMSF, 2 mg/mL pepstatin, 2 mg/mL leupeptin, and 1 mM orthovanadate at 4°C. Proteins were resolved by SDS-PAGE and transferred to PVDF membrane (PerkinElmer Life Sciences, Boston, MA). Membranes were exposed to primary antibodies against α-SMA (Sigma) and β-tubulin (PA5-16863, Thermo Scientific Rockford, IL, USA), and secondary antibodies coupled to horseradish peroxidase (KPL, Gaithersbury, MD, USA) and developed (ECL kit, Amersham Biosciences, Piscataway, NJ).

### Detection of senescence associated beta-galactosidase (SA-βgal)

GF were incubated for 3 days with 10% v/v serum from young, middle-aged and aged volunteers. Cells were washed in PBS and fixed with 2% w/v paraformaldehyde and 0,2% v/v glutaraldehyde for 5 minutes. Then, stained with solution 40 mM citric acid/Na phosphate buffer, 5mM K_4_[Fe(CN)_6_]*3H_2_O, 5mM K_3_[Fe(CN)_6_], 150 mM NaCl, 2mM MgCl_2_ and 1mg/mL X-gal (Bioline,Taunton, MA) during 24 hours [12] (Debacq-Chainiaux, *et al*, 2009). Nuclei were stained with Mayer´s Hematoxylin. As a positive control of cellular senescence, cells were exposed to 100μM H_2_O_2_ for 1 h and then treated with 10% FBS for 3 days.

#### Statistical analysis

All experiments were performed at least four times on separate occasions using cells from 5 different donors and at least 8 blood different donors. Statistical analysis was performed using the Mann-Whitney and Kruskal Wallis non-parametric tests. The statistical software Prism 5.0 from GraphPad was used (La Jolla, CA). In all analyses, p<0.05 was considered to indicate statistical significance.

## Results

### Impaired wound repair and proliferation in old rats

**Fig 1:**
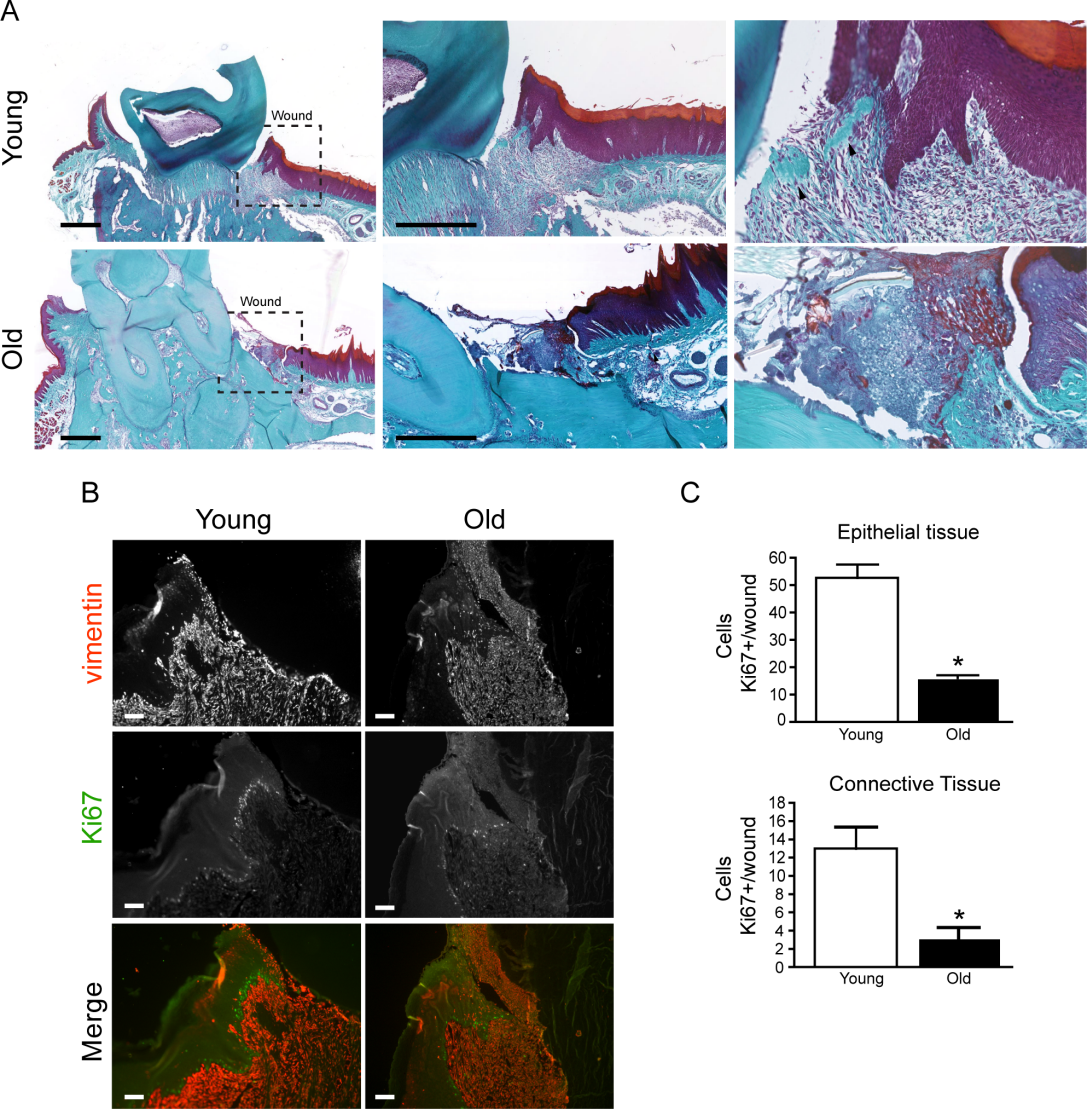
Old rats present impaired wound healing compared to young rats. Full-thickness wounds were made in the palatal gingiva. **A.** Sections of wounded gingiva from young and aged rats were stained with Masson Trichrome at day 7 post wounding. Images were scanned with nanozoomer at 2,5X, 5X and 20X. Scale bar:400μm. **B** Quantification of area granulation tissue in wounded area. **C** Immunostained for Ki67 (green) and vimentin (red). Scale bar 50μm **C** Quantification of positive cells for Ki67/total cells in epithelial and the connective tissue in the wound area. Arrowhead indicated focus of collagen. Bars indicate standard error. All assays were performed in quadruplicate. Asterisks indicate statistically significant differences (p = 0.0285 for Epithelial Tissue) and p = 0.00286 for Connective Tissue. n = 5.

Wound healing was evaluated after 7 days of gingival injury. We found a significant delay in wound closure in old rats (Fig 1A and 1B). Young rats show an increase in the area of granulation tissue compared with old rats (0.771± 0.037 versus 0.126 ±0.012 mm^2^.) Using the Masson trichrome staining we determined that collagen filled 41% and 2.1% of the lesion of young and aged wounds respectively. Moreover, we observed an increase of cellularity (nuclei in purple color at the connective tissue) in young rats (Fig 1A). We also evaluated whether changes in cell proliferation, measured through Ki67 staining, were associated with these responses. We found an increase of positive cells in young rats compared to old rats (52.75 versus 16.25 cells). Moreover, we found a 3 fold increase of positive cells for ki67 staining in the connective tissue when we compared young wounds to their aged counterparts, ranging the counts of cells from 13 cells in young rats versus 3.25 cells in old rats (Fig 1C). Vimentin, a cell marker of intermediate filament was used to identify mesenchymal cells (Fig 1C).

### Serum from middle-aged and aged donors do not induce cell proliferation in GF

**Fig 2:**
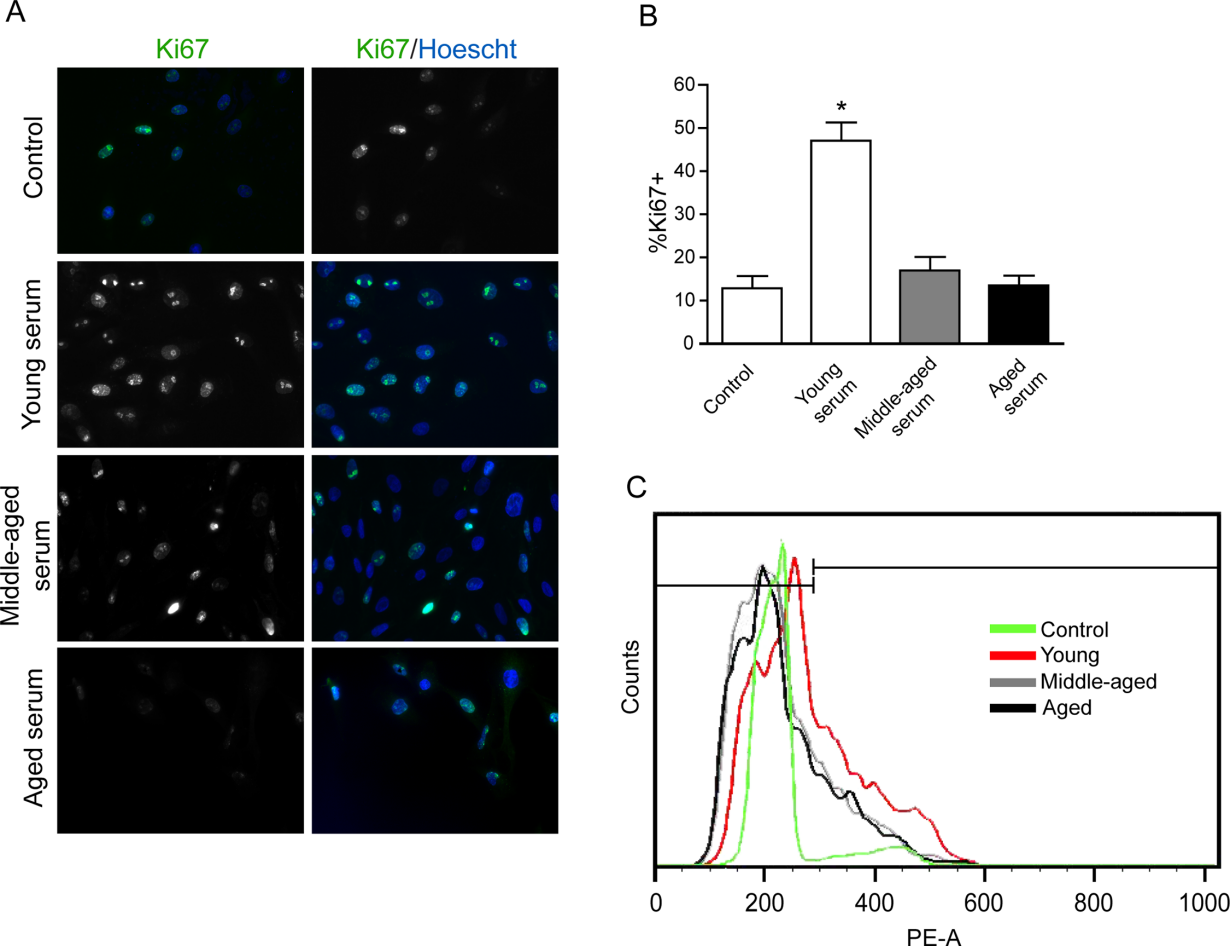
Middle-aged and aged serum do not induces GF proliferation. **A** Representative images of Immunofluorescence for Ki67 and Hoechst, GF treated with young serum (18 yo), middle-aged serum (30 yo) and aged serum (65 yo). **B** Graph indicated the quantification of ki67(+)cells/total cells. **C** Flow cytometry analysis showed that young serum promotes the proliferation of GF, graph of PI versus count of cells, control (green), young serum (red), middle-aged serum (gray) and aged serum (black). Asterisks indicate statistically significant differences between control and young serum treatment p = 0.001. Bars indicate standard error. All assays were performed in quadruplicate. (n = 8 young, n = 8 middle-aged, n = 8 aged).

We next determined whether cell proliferation defects detected in aged rats were related to serum derived-factors. Therefore, gingival fibroblasts derived from young subjects were exposed to blood serum obtained from the three age groups under study. GF were synchronized for 16h in DMEM without serum and incubated with 10% blood serum obtained from young (18–22 yo) middle-aged (30–48 yo) and aged (50–65 yo) volunteers for 24 h. As shown in Fig 2B the proportion of Ki67 positive/nuclei were 47.02 ± 4.31%, 16.93 ± 3.24%, 13.48 ± 2.28% for the young, middle-aged and aged groups respectively. We next performed a more specific assay to evaluate the proliferative response of cells. Evaluation of the proportion of cells in the S phase of the cell cycle determined that 8.85 ± 1.7%, 36.6 ± 1.54%, 24 ± 1.7%, 20.5 ± 1.32% were present in the control, young, middle-aged and aged group respectively. Fig 2C shows a representative curve of GF treated with one serum for each group. These results show that the increase in cell proliferation depends on serum donor age, whereas the incubation with young serum increased cell proliferation.

### Serum from middle-aged and aged donors stimulate cellular senescence by expression of SA-βgal

**Fig 3:**
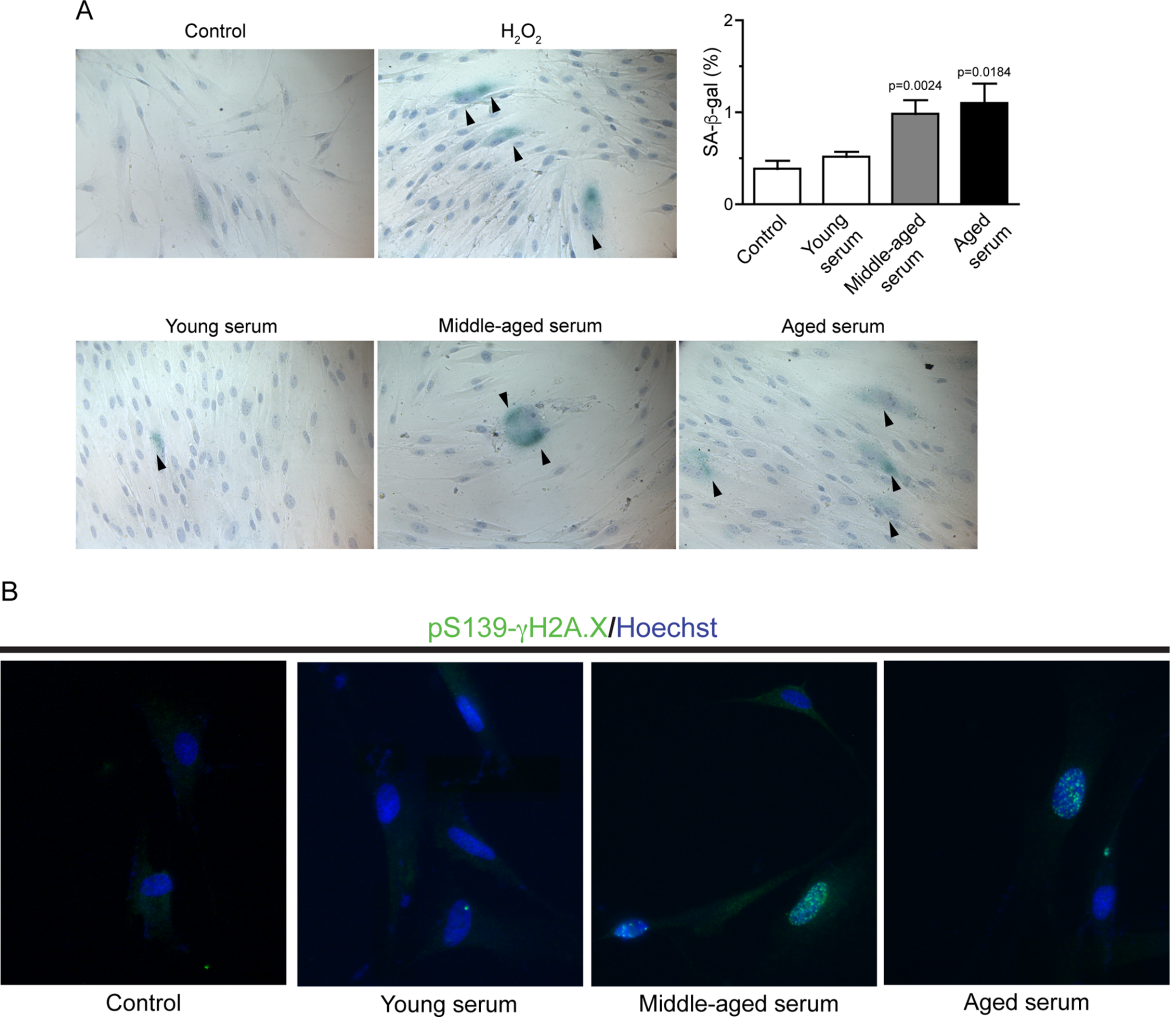
Middle-aged and aged serum induces some senescent cells. **A** Representative images of SA-β of control cells, cells treated with 100μM H_2_O_2_, 10% v/v young, middle-aged and aged serum. Graph shows the quantification of SA-βgal positive cells versus total cells stained with Mayer´s Hematoxylin. Arrow head indicated positive cell for SA-β. **B** Immunofluorescence of histone H2A.X phosphorylated at Ser139 and DNA stained by Hoechst. All assays were performed in quadruplicate (n = 8 young, n = 8 middle-aged, n = 8 aged).

Salient features of the senescent cells are, the arrest of cell cycle, increased in size and the expression a senescent–associated β galactosidase (SA-βgal) which partly reflects the increase in lysosomal mass senescence growth [13]. We evaluated whether serum from different ages might contain factors that may promote the development of cellular senescence. Therefore, GF were incubated with 10% serum from different ages during 3 days. The frequency of senescent cells in culture (positive for SA-βgal) increased when cells were exposed to serum from aged subjects ranging from 0.38 ± 0.21% (10% FBS;control), 0.51 ± 0.30% (young serum), 0.98 ± 0.55% (middle-aged serum), 1.15 ± 0.52% (aged serum) or positive control (6.5 ± 1.1%; 100μM H_2_O_2_) (Fig 3). Moreover, we analyzed the distribution of one of the major trigger of cell senescence, phosphorylation of Ser139 of histone H2A.X [14]. Using this cell marker we observed an increase level in γ-H2A.X in cells treated with middle-aged and ages serum (Fig 3B). In S2 Fig we show significantly increases of γ-H2A.X foci-positive cells, presumably fibroblast in the tissue close to the wound in old rats than in young tissue.

### Serum from middle-aged and aged donors do not stimulate myofibroblastic differentiation

**Fig 4:**
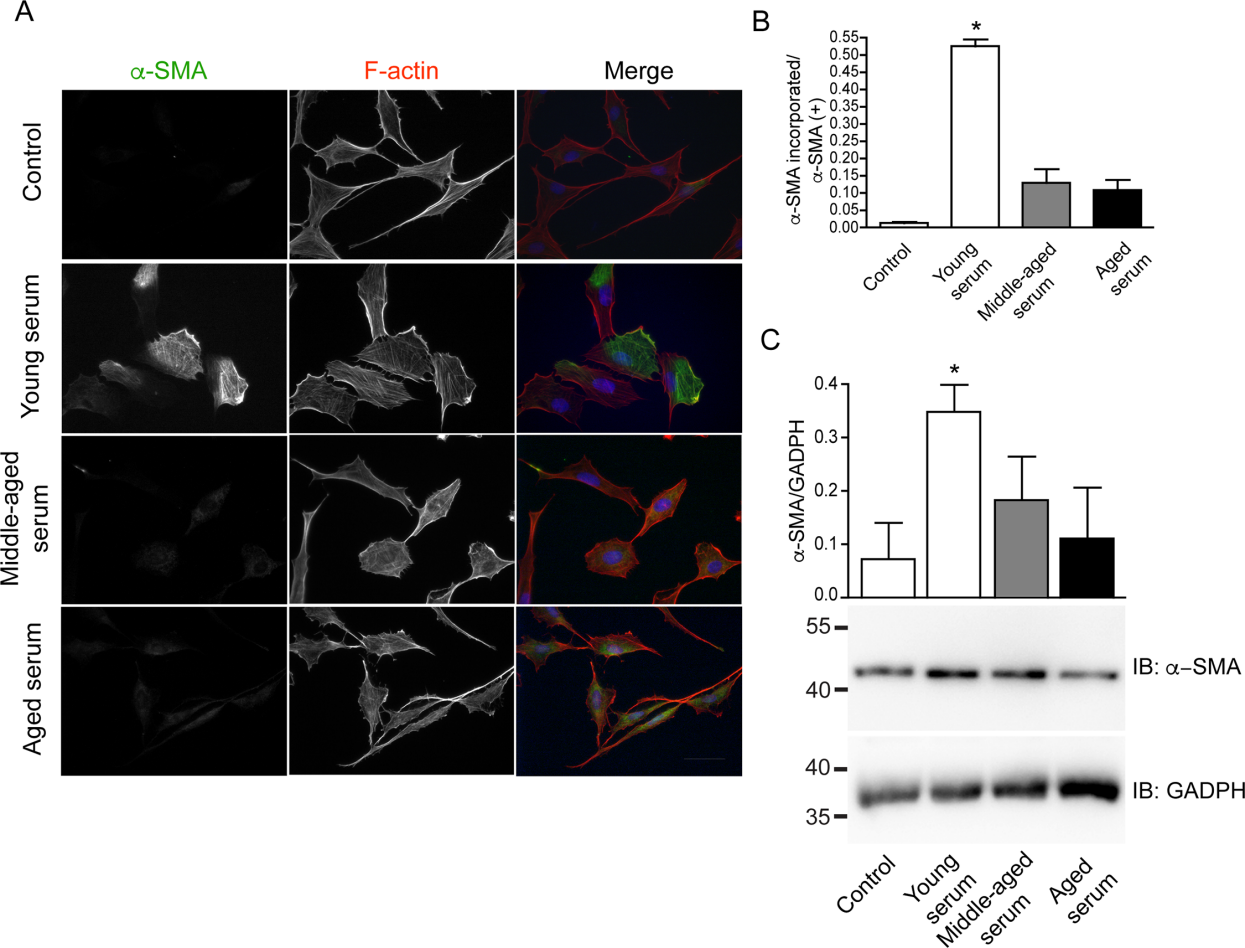
Young serum induces differentiation to myofibroblast with α-SMA incorporated into stress fiber. **A** Immunofluorescence of α-SMA (green), F actin (red) and DNA with Hoechst (blue) of GF treated during 72 hours with 10% young serum, middle-aged serum and elderly serum. **B** Graph indicated the quantification of α-SMA incorporated into stress fibers/ α-SMA (+) cells. Asterisks indicate statistically significant differences between control and young serum treatment p = 0.0121 (n = 9 young, n = 9 middle-aged, n = 8 aged) in 4 different experiment. **C** Serum-starved cells were stimulated with serum from different ages for 72 h, α-SMA and GAPDH were analyzed through Western-blot of the cell lysates (n = 7 young, n = 7 middle-aged, n = 6 aged). Bars indicate standard error. Asterisks indicate statistically significant differences (*p = 0*.*0356*). Scale bar 10μm.

Myofibroblasts play a fundamental role in collagen secretion and matrix remodeling [6]. Therefore, we evaluated whether myofibroblastic differentiation was affected by serum derived from different donors. GF were treated with 10% serum from different ages during 72 h. Using, immunofluorescence we observed that in GF treated with young serum, α-SMA was distributed in association with actin stress fibers (Fig 4). However, α-SMA was not incorporated into the stress fiber in cells treated with middle-aged and aged serum (Fig 4A). This response was quantified in the graph shown in Fig 3B, demonstrating a significant increase in the proportion of cells with α-SMA incorporated into actin stress fibers in cells exposed to serum from young donors. Furthermore, the total α-SMA protein levels were increased in cell lysates exposed to young serum were compared to control, middle-aged or aged serum (Fig 4C).

### Human blood factors related to wound healing change during aging

**Fig 5:**
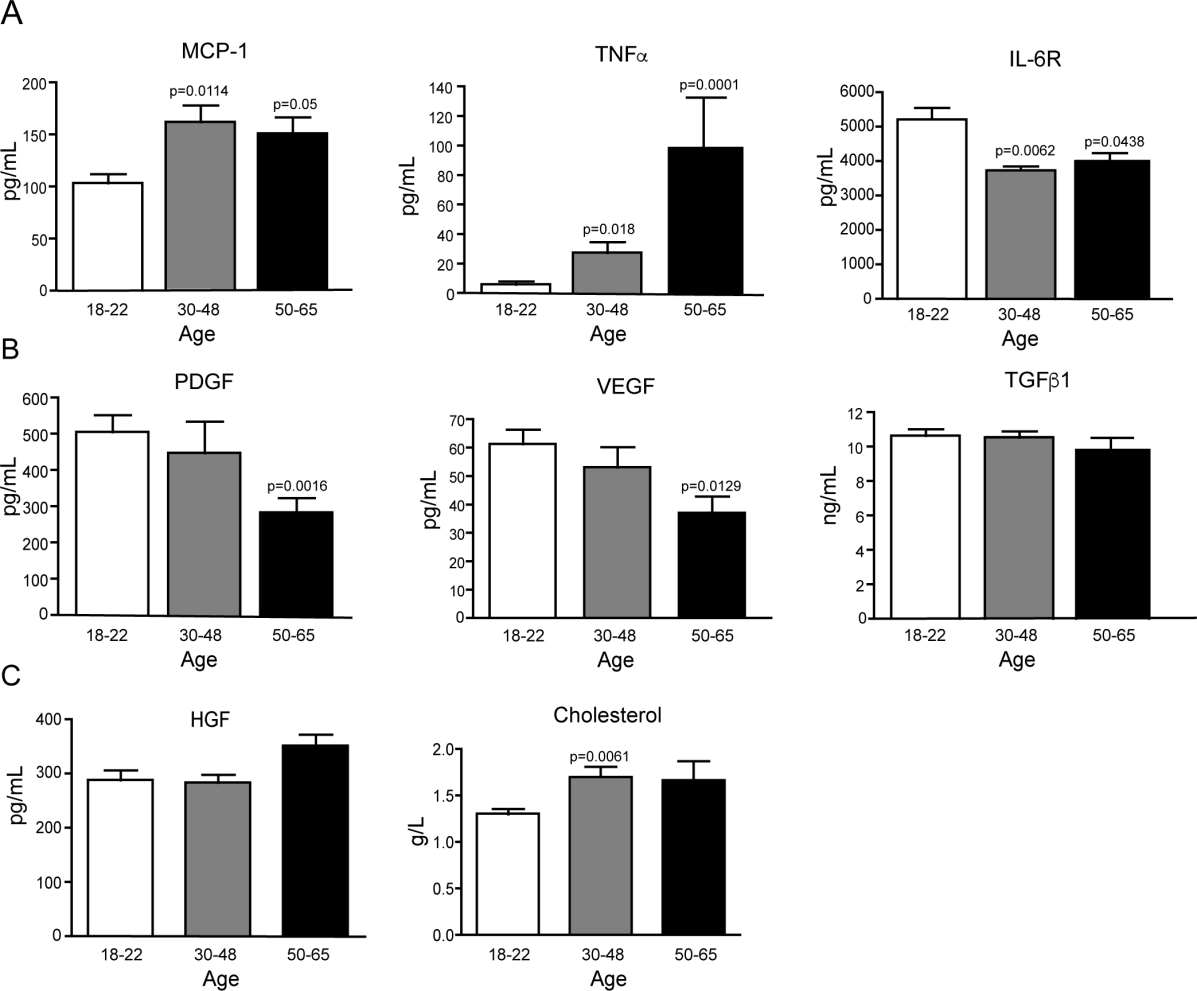
Blood soluble factors related to wound healing vary with the age. **A** Quantification of MCP-1, TNF and IL-6R.**B** Quantification of growth factors PDGF-BB, VEGF, TGFβ1 and HGF. **C** Graph of the levels of total cholesterol. Asterisks indicate statistically significant differences. Young (n = 21), over 50 yo (n = 10). The group in between ages was middle-aged 30–48 yo (n = 13).

We then hypothesized whether serum from subjects of different ages may show differences in the concentration of cytokines and growth factors that may explain the above-described responses. Therefore, MCP-1 and IL6R protein levels were evaluated in the serum of these volunteers. Monocyte Chemoattractant Protein-1 (MCP-1) is a chemokine that regulates the migration and infiltration of monocytes, memory T lymphocytes and natural killer (NK) [15]. Soluble IL-6 receptor (IL-6R) forms a ligand-receptor complex with IL-6 to stimulate proliferation, differentiation and inflammatory processes [16]. Serum concentration of MCP-1 was found to increase with age from 108.55 ± 8.85 pg/mL (young) 152.78 ± 13.35 pg/mL (middle-aged) to 155.87 ± 13.54 pg/mL (aged). Whereas IL-6R was found at decreased levels in serum of aged subjects ranging from 4981 ± 392 pg/mL (young), 3740 ± 110.7 pg/mL (middle-aged), 4005 ± 234.5 pg/mL (aged serum). Also, Tumor Necrosis Factor alpha (TNF) a potent inflammatory cytokine expressed during the inflammatory phase of wound healing [17]. We found an increase in TNF protein levels with increasing age with values ranging from 5.92 ±1.78 pg/mL (young serum), 27.52±5.6 pg/mL (middle-aged) and 98.29 ± 20.6 pg/mL (aged serum) (Fig 5A). We next evaluated growth factors involved in cell proliferation [18] and angiogenesis in the granulation tissue such as Platelet Derived Growth Factor (PDGF) and Vascular Endothelial Growth Factor (VEGF). We found that PDGF decreased with the age from 507.76 ± 38.78 pg/mL (young serum), 447.4 ± 54.8 (middle-aged) and 283.68 ± 27.36 pg/mL (aged serum). VEGF levels decreased with the age from 61.259 ± 5.3 pg/ml (young serum) to 53.097 ± 6.99 pg/mL (middle-aged serum) and 37.01± 5.39 pg/mL (aged serum). In addition we evaluated Transforming Growth Factor beta 1 (TGFβ1), responsible for myofibroblastic differentiation. Surprisingly, we did not found differences in the serum levels for this factor with the following values for young (10.64 ± 2.3 ng/mL), middle-aged (10.55 ± 1.53 ng/mL) and aged (9.81 ± 2.313 ng/mL) samples (Fig 5B). We finally evaluated Hepatocyte Growth Factor, not finding significant differences between these groups, 288.63 ±16.8 for young serum, 283.6 ± 8.15 for middle-aged and 351.5 ± 10.121 pg/mL for aged group. These results confirm that not all growth factors were modulated by age. Cholesterol levels measured as a control, were detected at increased levels in middle aged subjects when compared to young serum, but in average all ages were less than 2 g/L (Fig 5C).

### Serum from old rat present inflammatory cytokines

To evaluate the presence of pro inflammatory cytokines in rat blood serum we realize a proteome profile cytokine antibody array, in which old blood serum have higher levels of CINC-1, IL-1α, SICAM, CX3CL1, MIP-1α compared to young serum. LIX and RANTES were found at similar levels. Unfortunately, we could not detect TNF by this method.

Similar to human blood serum from middle aged and aged, blood serum from old rats contain several cytokines related to inflammation like MIP1α that is a member of the C-C family of chemokines that exhibit a variety of proinflammatory activities and macrophages are a rich source of MIP1alpha [19], also contain cytokine-induced neutrophil chemoattractant 1 (CINC-1) that play critical roles as a mediator of inflammatory reactions with neutrophil infiltration in rats [20] and IL-1α that is a potent inflammatory cytokine that activate the inflammatory process. [21].

**Fig 6:**
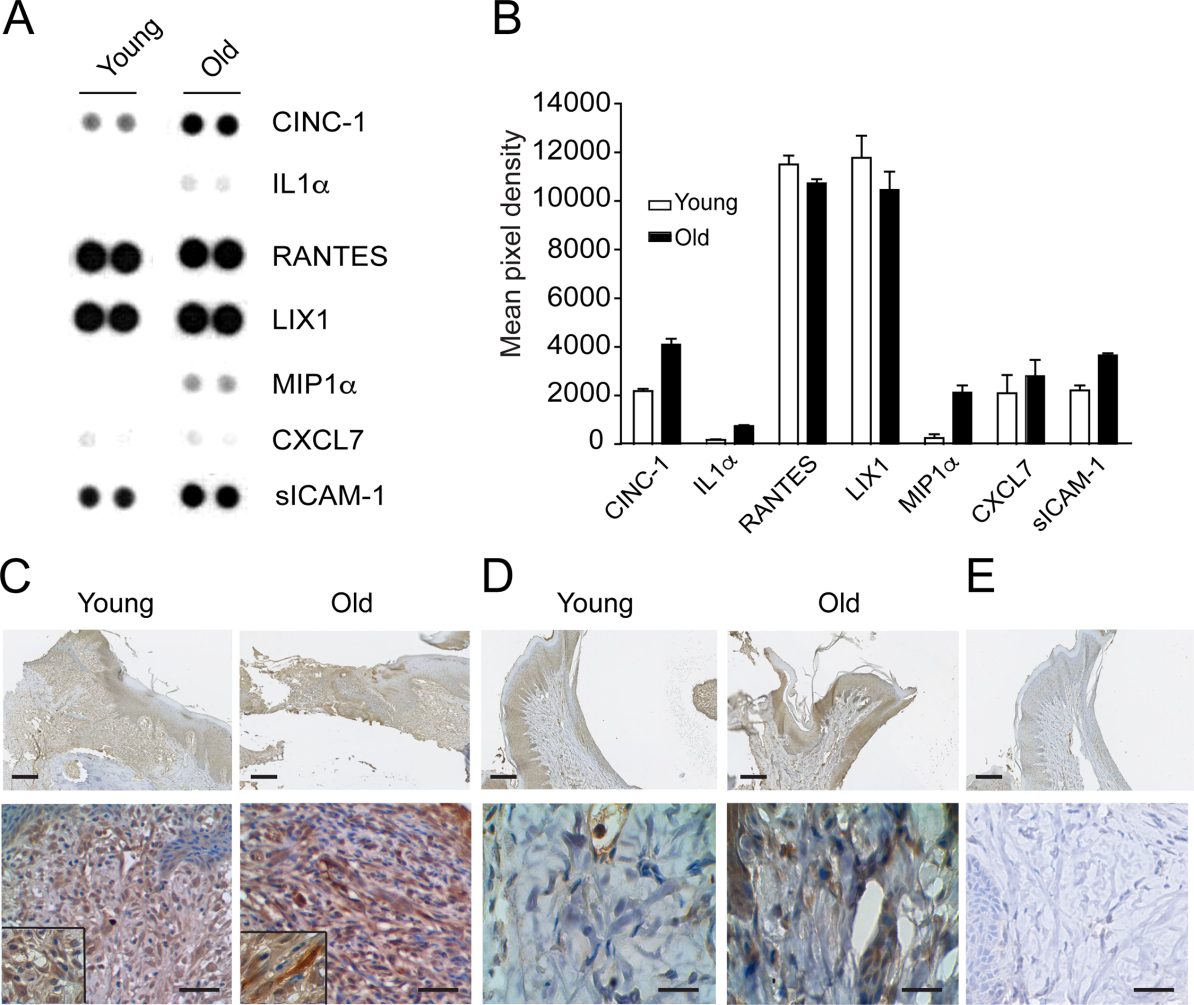
Old rat serum present higher levels of pro-inflammatory cytokine. **A** Representative image of the cytokine proteome array from young and old rat serum **B** Graph indicated the quantification of the pixel mean density of each cytokine. **C** TNF distribution in wound from young and old rats after 7 days of repair 5X, 40X and inset of 100X.**D** TNF distribution in non wound gingiva 5X (scale bar 150 um) and 40X (scale bar 50um). **E** Negative control 5X and 40X.

One of the cytokine that increased in human blood serum during aging is TNF. We evaluated locally the expression of TNF in wound from young and old rats (Fig 6C). Wound from old rats present higher staining of TNF compared to wound from young rats (Fig 6C). Moreover, healthy gingiva (non injury) shows less staining compared to a wound site (Fig 6D), suggesting an important role of TNF during wound healing. Using the software NDP analysis, we observed that the minimal intensity of the selected ROI in wound from young rats was 63.6 ± 3 versus 92 ±3.5 in old rats. Moreover, the mean intensity in young rats was 122 versus 138 founded in old rats. This analysis suggests that the expression of TNF was higher in old rats compared to young rats.

Interestingly, young gingival fibroblast treated with 10% young serum and 300 pg of TNF during 3 days, show that the addition of TNF to young serum increased of cellular senescence as shown in S1 Fig.

The identification of factors enriched in young blood or lost in old blood that can improve wound healing is a key research goal.

## Discussion

The findings of the present study allow us to propose that factors found in the serum of middle-aged and aged subjects may contribute to delayed wound healing by decreasing cell proliferation and myofibroblastic differentiation and by stimulating cellular senescence. All these responses may impact wound healing since they are necessary to augment the amount of cells needed for tissue repair and for the secretion and organization of collagen in tissues [5]. Moreover, senescent cells have been associated with impaired wound healing as well [22]. Therefore, serum derived factors might be responsible, at least in part, for delayed wound healing responses associated with aging.

Our study suggests that aging may have an early manifestation in serum related to the variations in some inflammatory cytokines and growth factors. These changes included an increase in MCP-1 and TNF in the serum of aged subjects evaluated in this study and a decrease in PDGF, VEGF and IL-6R. In addition, no changes were found in TGFβ1 and HGF protein levels. According to these findings, the augmented levels of inflammatory cytokines might contribute with increased levels of inflammatory cells in tissues that may delay wound repair. It is important to consider that TGFβ-stimulated myofibroblastic differentiation is effectively inhibited by the inflammatory cytokine TNF as described for dermal [17] and gingival fibroblast [23] In addition, TNF may impair wound healing in diabetes [24], suggesting that this cytokine is probably implicated in delayed wound healing in different contexts. The results described in our study suggest that increased TNF levels found in serum from middle-aged and aged donors may be responsible for the decrease in myofibroblastic differentiation found in cells exposed to these factors. More studies are needed to identify and quantify the amount of TGFβ1, TNF and other relevant cytokines during wound healing in young and aged tissues.

The diminished levels of PDGF and VEGF found in the serum of middle-aged and aged subjects might also contribute to explain the reduction in cell proliferation observed in our study. PDGF and VEGF are also involved in cell migration [25] and angiogenesis [25]. Although we did not test this possibility in the present study, these assays should identify other relevant functions putatively modulated by serum factors in aged subjects.

Previous studies have evaluated PDGF, VEGF and TGFβ1 serum levels in 18 years old males. These determinations were similar to our findings with measurements approaching 1.7 ng/mL for PDGF, 20 ng/mL for TGFβ1 and 91.83 pg/mL of VEGF [26,27]. Moreover, a similar result was obtained by Bayer *et al*, (2012), who found that TGFβ1 serum levels did not change with age [28]. Moreover, work by Weibrich et al (2002) analyzed platelet rich plasma of 203 donors between 17 and 62 years old and found no influence of gender or age on platelet count [29].

In recent studies, Belsky *et al* (2015) analyzed the correspondence between chronological and physiological ages. In that study they evaluated young adults of 38 years old, showing a great discrepancy between chronological age and biological age [30]. These results coincide with our observations concerning the concentration of soluble mediators present in serum in the middle-aged group. Further studies are needed to better characterize aging within this group.

It has been proposed that senescent cells develop the so called senescent associated secretory phenotype (SASP), a trait characterized by the production of growth factors, matrix remodeling enzymes and inflammatory cytokines that include IL-6 and IL-8 among others [13]. In this work we have observed MCP-1 and TNF are found at increased levels in the serum of aged subjects. It is possible to propose that these inflammatory cytokines may be responsible for the detrimental effects of middle-aged and aged serum on cell proliferation and myofibroblastic differentiation. It is interesting to consider whether cells responsible for the secretion of these cytokines are involved cellular senescence at some level. Moreover, our observation of the effect of middle-aged and aged serum on the induction of cellular senescence in young fibroblasts may also suggest that serum may contribute to the initiation of cellular senescence in tissues. It is important to consider that in aged organisms the proportion of senescent cells may vary between 1% to 15% [31]. Hubackova *et al* (2016) demonstrated that TNFα and IFN gamma induce cellular senescence in mouse tumor cells [32]. Given our observation of increased TNF levels in the serum of aged subjects it is interesting to propose whether this cytokine may play a role in the induction of cellular senescence in periodontal tissues.

Interestingly, diabetes delay wound healing, which may result in limb amputation. Enhanced levels of advanced glycation end product (AGEs) and TNF are associated with these phenomena. Impaired wound healing in diabetic animals are improved by blocking TNF, which reduces infiltration by proinflammatory macrophages, decreases fibroblast apoptosis and improves collagen synthesis [33].

Wang et al demonstrates that γ-H2A.X immunohistochemistry is a reliable quantitate indicator of senescence in fibroblast. Moreover, increased with age in dermis, liver and gut epithelium [14]. In our data, we show increased levels of γ‒H2A.X foci-positive cells in old rats compared to young rats (S2 Fig).

The present study provides a series of observations that contribute to understand the deficiencies detected in gingival wound healing during aging and suggests that it is important to study middle-aged subjects during the early manifestations of tissue changes associated with aging.

## Supporting information

S1 Fig TNF increased cellular senescence. (DOCX)

S2 Fig Old rats have an increased of γ-H2A.X positive cells. (DOCX)

S1 File Blood samples. (DOCX)
