# Correction: Aged blood factors decrease cellular responses associated with delayed gingival wound repair

**DOI:** 10.1371/journal.pone.0189566

**Published:** 2018-01-23

**Authors:** The PLOS ONE Staff

The figure files for S1 Fig and S2 Fig are omitted from the list of Supporting Information. The publisher apologizes for the errors. The two figures and their corresponding captions can be viewed below.

## Supporting information

S1 Fig TNF increased cellular senescence. Representative images of SA-β of control cells, cells treated with 150, 300 and 500 pg/mL of TNF, 10% v/v young serum and 10% v/v young serum plus 300 pg/mL TNF. Inset of the flat and enlarged morphology characteristic of the senescent cells. Graph shows the quantification of percentage SA-βgal positive cells. 20X. * Indicate statistically significant differences to 0 pg of TNF. ** Indicates statistically significant differences between Young serum and Young serum complemented with 300pg of TNF.
(TIF)

S2 Fig Old rats have an increased of γ-H2A.X positive cells. **A** Wound gingiva of 2 and 18 years old rats were stained for γ-H2A.X. Examples of connective tissue of the wound are shown. Scale bar 50 μm. 63X. **B** Quantification of positive cells for γ-H2A.X versus total cells.
(TIF)
