# Supplementary material for: Aged blood factors decrease cellular responses associated with delayed gingival wound repair
Source: PLoS One. 2017 Sep 12;12(9):e0184189. doi: 10.1371/journal.pone.0184189 (PMC5595322; doi:10.1371/journal.pone.0184189)
Supplement: S1 Fig — (DOCX) [file pone.0184189.s001.docx]

# Supporting information

# S1 Fig. TNF increased cellular senescence.

# Representative images of SA-β  of control cells, cells treated with 150, 300 and 500 ng/mL of , 10% v/v young serum and 10% v/v young serum plus 300 μg/mL TNF. Inset of the flat and enlarged morphology characteristic of the senescent cells. Graph shows the quantification of percentage SA-βgal positive cells. 20X. * Indicate statistically significant differences to 0 pg of TNF. ** Indicates statistically significant differences between Young serum and Young serum complemented with 300pg of TNF.
