# Supplementary material for: Aged blood factors decrease cellular responses associated with delayed gingival wound repair
Source: PLoS One. 2017 Sep 12;12(9):e0184189. doi: 10.1371/journal.pone.0184189 (PMC5595322; doi:10.1371/journal.pone.0184189)
Supplement: S2 Fig — (DOCX) [file pone.0184189.s002.docx]

# S2 Fig. Old rats have an increased of γ-H2A.X positive cells.

# Wound gingiva of 2 and 18 years old rats were stained for γ-H2A.X. Examples of connective tissue of the wound are shown. Scale bar 50 μm. 63X. B Quantification of positive cells for γ-H2A.X versus total cells.
