# Supplementary material for: Aged blood factors decrease cellular responses associated with delayed gingival wound repair
Source: PLoS One. 2017 Sep 12;12(9):e0184189. doi: 10.1371/journal.pone.0184189 (PMC5595322; doi:10.1371/journal.pone.0184189)
Supplement: S1 File — (DOCX) [file pone.0184189.s003.docx]

# S1 File. Blood samples

# The research team did an open invitation to members from Hospital Clínico, Faculty of Medicine and Faculty of Dentistry from Universidad de Chile. Enrollment occurred between November 2015 and January 2017. Eligible volunteers were healthy men >18 years old. Volunteers were considered ineligible in case of a documented medical history of 1) Chronic inflammatory diseases; 2) Recent infection (<6 months); 3) Primary immunodeficiency; 4) Immunosuppression treatment; 5) Recent surgery (< 12 months); 6) Recent trauma (<6 months); 7) Hematologic pathologies; 8) Cancer in the past 5 years; 9) Active smoking; 10) Obesity. Clinical history and health-related habits were obtained by an interview performed by an anesthesiologist from Hospital Clínico Universidad de Chile, in order to rule out these conditions and obtain additional epidemiological data. None of the donors were obese, average of weight young, (73.8 ±10.12 kg) middle-aged (79.4 ± 5.7 kg), aged (82 ±8.48 kg) and the IMC were young, (23.79) middle-aged (26.42), aged (26.2). All samples were obtained from a peripheral vein in the arm and the serum was stored at -80°C.
