# Supplementary figures and images for: Correction: Aged blood factors decrease cellular responses associated with delayed gingival wound repair
Source: PLoS One. 2018 Jan 23;13(1):e0189566. doi: 10.1371/journal.pone.0189566 (PMC5779646; doi:10.1371/journal.pone.0189566)

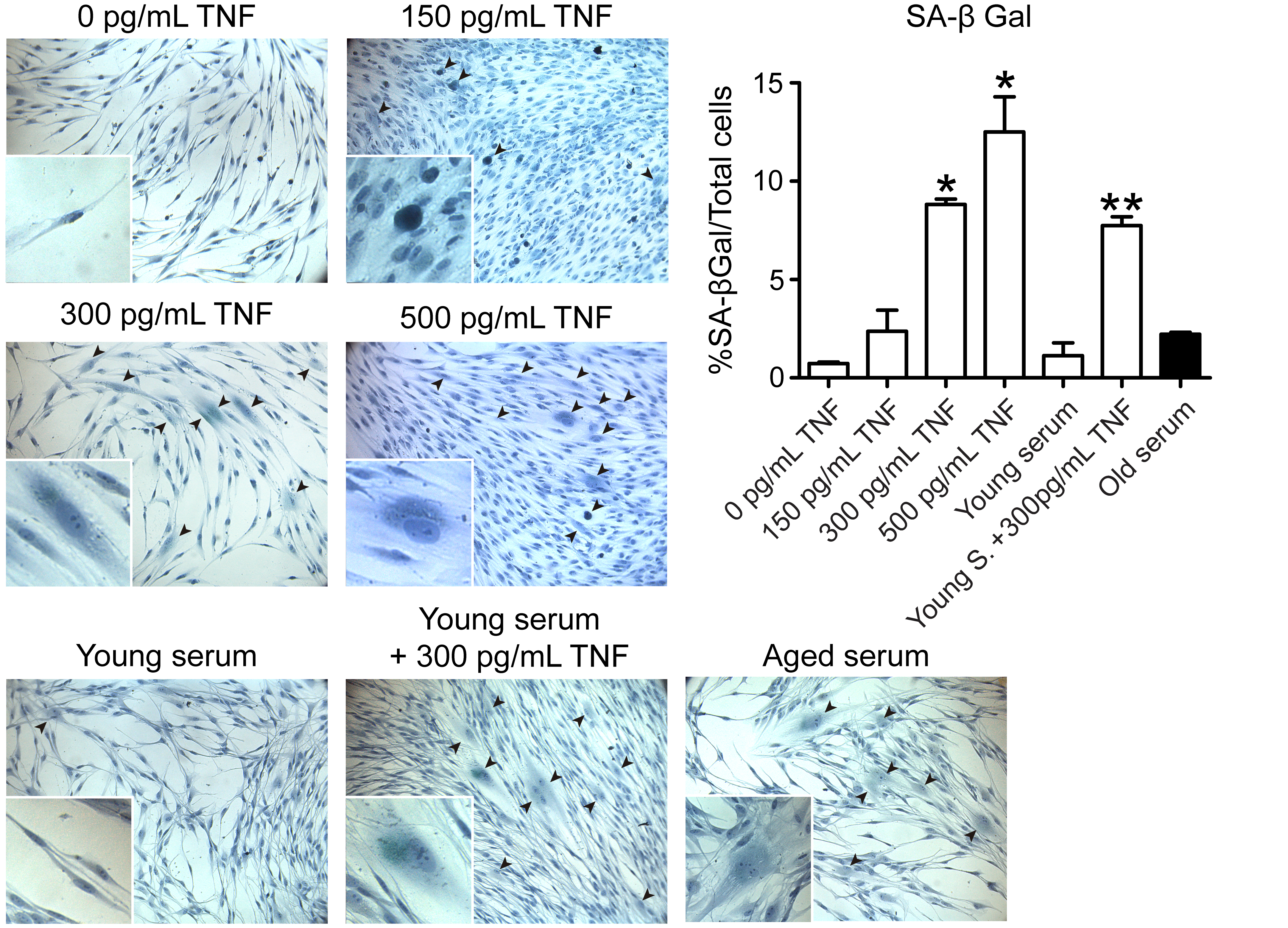

Supplement: S1 Fig — Representative images of SA-β of control cells, cells treated with 150, 300 and 500 pg/mL of TNF, 10% v/v young serum and 10% v/v young serum plus 300 pg/mL TNF. Inset of the flat and enlarged morphology characteristic of the senescent cells. Graph shows the quantification of percentage SA-βgal positive cells. 20X. * Indicate statistically significant differences to 0 pg of TNF. ** Indicates statistically significant differences between Young serum and Young serum complemented with 300pg of TNF. (TIF) [file pone.0189566.s001.tif]

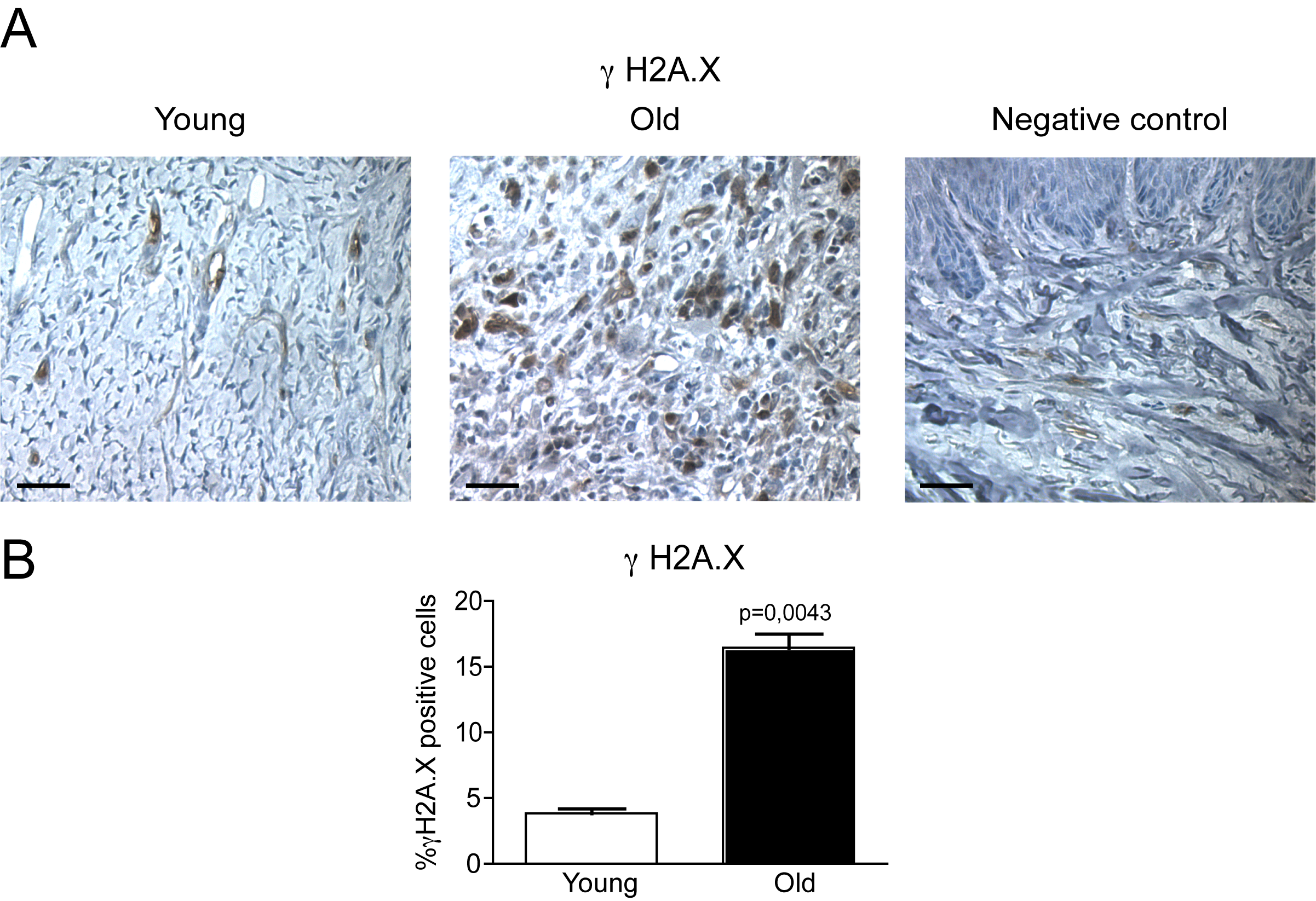

Supplement: S2 Fig — A Wound gingiva of 2 and 18 years old rats were stained for γ-H2A.X. Examples of connective tissue of the wound are shown. Scale bar 50 μm. 63X. B Quantification of positive cells for γ-H2A.X versus total cells. (TIF) [file pone.0189566.s002.tif]
